# Reduced Mitochondrial DNA Content Associates with Poor Prognosis of Prostate Cancer in African American Men

**DOI:** 10.1371/journal.pone.0074688

**Published:** 2013-09-23

**Authors:** Shahriar Koochekpour, Timothy Marlowe, Keshav K. Singh, Kristopher Attwood, Dhyan Chandra

**Affiliations:** 1 Department of Cancer Genetics, Roswell Park Cancer Institute, Buffalo, New York, United States of America; 2 Department of Urology, Roswell Park Cancer Institute, Buffalo, New York, United States of America; 3 Department of Pharmacology and Therapeutics, Roswell Park Cancer Institute, Buffalo, New York, United States of America; 4 Departments of Genetics, Pathology, and Environmental Health, UAB Comprehensive Cancer Center and Center for Free Radical Biology, School of Medicine, University of Alabama, Birmingham, Alabama, United States of America; 5 Department of Biostatistics and Bioinformatics, Roswell Park Cancer Institute, Buffalo, New York, United States of America; University of Dayton, United States of America

## Abstract

Reduction or depletion of mitochondrial DNA (mtDNA) has been associated with cancer progression. Although imbalanced mtDNA content is known to occur in prostate cancer, differences in mtDNA content between African American (AA) and Caucasian American (CA) men are not defined. We provide the first evidence that tumors in AA men possess reduced level of mtDNA compared to CA men. The median tumor mtDNA content was reduced in AA men. mtDNA content was also reduced in normal prostate tissues of AA men compared to CA men, suggesting a possible predisposition to cancer in AA men. mtDNA content was also reduced in benign prostatic hyperplasia (BPH) tissue from AA men. Tumor and BPH tissues from patients ≥60 years of age possess reduced mtDNA content compared to patients <60 years of age. In addition, mtDNA content was higher in normal tissues from patients with malignant T3 stage disease compared to patients with T2 stage disease. mtDNA levels in matched normal prostate tissues were nearly doubled in Gleason grade of >7 compared to ≤7, whereas reduced mtDNA content was observed in tumors of Gleason grade >7 compared to ≤7. Together, our data suggest that AA men possess lower mtDNA levels in normal and tumor tissues compared to CA men, which could contribute to higher risk and more aggressive prostate cancer in AA men.

## Introduction

In terms of diagnosis and mortality, prostate cancer is one of the leading cancers among American men. Increased prostate cancer-related diagnoses and mortality are commonly associated with African American (AA) men compared to Caucasian American (CA) men [1]–[3]. Although diet and family history likely play important roles in this disparity, genetic factors may also be key factors influencing prostate tumorigenesis. Mutation or deletion of mitochondrial DNA (mtDNA) has been associated with multiple cancers including prostate cancer [4]–[6]. *In vitro* and *in vivo* analyses demonstrate that reduction of mtDNA content is associated with acquisition of an androgen-independent phenotype and leads to prostate cancer progression [6]–[8]. Reduced level of mtDNA and mutations in mtDNA have been reported in multiple cancer types such as prostate [9], [10] breast [11], renal [12], and liver [13]. In contrast, increased mtDNA content has also been reported in other types of cancer including prostate cancer [14]–[16]. Depletion or reduction of mtDNA inhibits apoptosis, thereby contributing to cancer progression [17]–[19]. In addition, depletion of mtDNA leads to the development of resistance to anticancer agents, including cisplatin and adriamycin [20]. However, increased mtDNA levels are also associated with acquired resistance to docetaxel in head and neck cancer cells [21]. Hence, maintaining a steady-state level of mtDNA in cancer cells is critical for a positive outcome during cancer therapy.

How mtDNA regulates the tumorigenesis process is not clearly defined, but current evidence suggests that mutation, reduction, or deletion of mtDNA lead to defective oxidative phosphorylation (OXPHOS), increased reactive oxygen species (ROS) production, induction of the glycolytic pathway, increased expression of prosurvival proteins, which ultimately results in cancer proliferation and tumorigenesis [19], [22]–[24]. Therefore, modulation of mtDNA content in cancer, including prostate cancer, is significant to understanding the disease process. Although imbalanced levels of mtDNA have been reported in prostate cancer [7], [16], [24]–[26], differential levels of mtDNA in AA and CA prostate cancer patients have not yet been reported. Here, we provide the first evidence that reduced mtDNA content may contribute to the higher incidence as well as aggressive prostate cancer phenotype in AA men compared to CA men.

## Results

### BPH and Prostate Tumor Tissues have Reduced Levels of mtDNA

**Figure 1:**
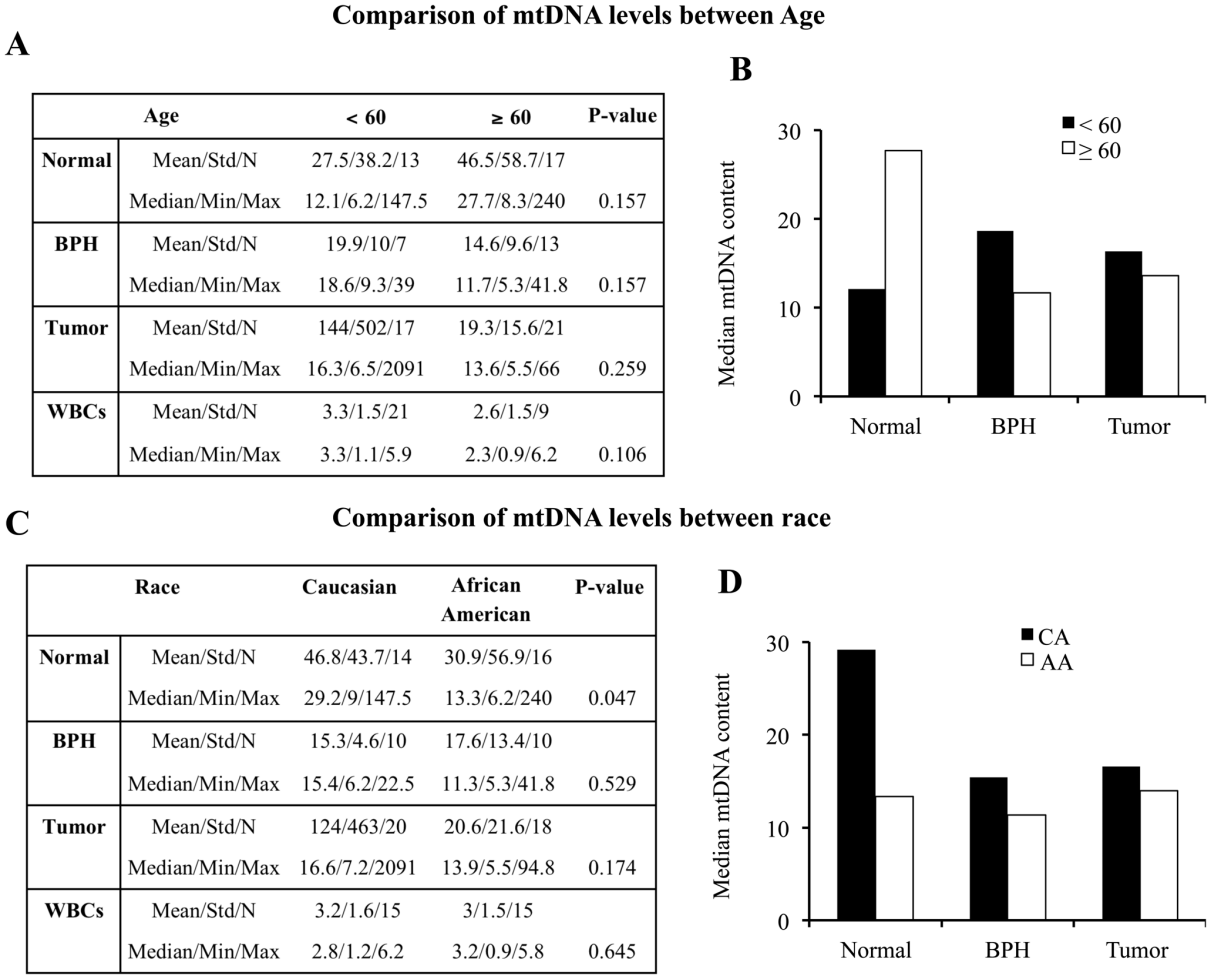
Comparison of mtDNA content between age and race. A, mtDNA content was compared by age using Wilcoxon rank-sum test. B, Median mtDNA content in normal, BPH, and tumors from patients <60 and ≥60 years of age are plotted for comparison. C, mtDNA content was compared by race using Wilcoxon rank-sum test. D, Median mtDNA content in normal, BPH, and tumors from AA and CA patients are plotted for comparison.

**Figure 2:**
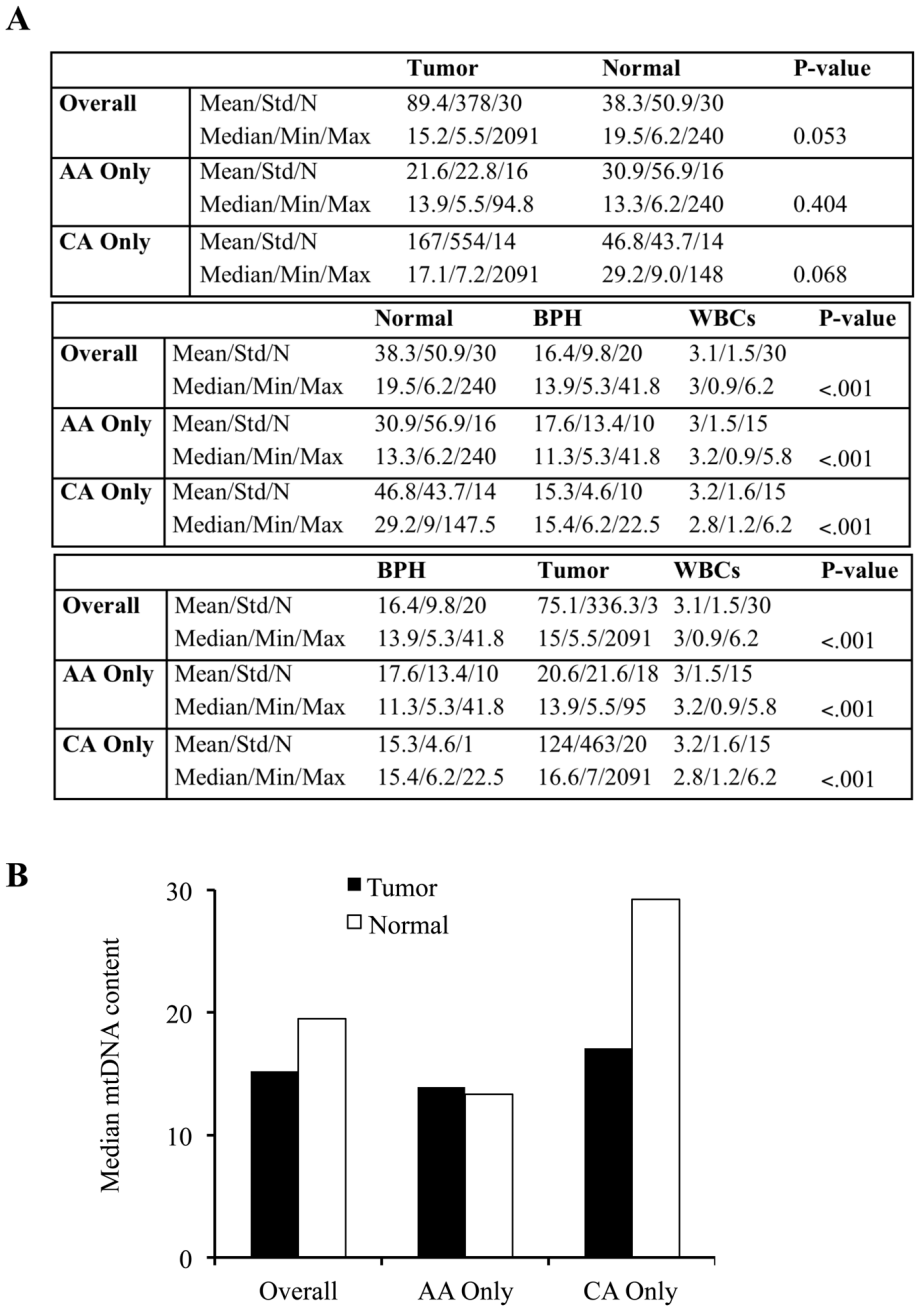
Comparison of mtDNA content in normal, BPH, WBC, and tumors from AA and CA prostate cancer patients. A, Due to paired nature of data, median mtDNA values were compared between tumor and normal samples using Wilcoxon signed-rank tests (top panel of table). Normal, BPH, and WBCs were compared using Kruskal-Wallis tests (middle panel). Tumor, BPH, and WBCs were compared to one another using Kruskal-Wallis tests (lower panel). B, Median mtDNA values from top table of panel A are plotted for comparison.

OXPHOS defects are one of the hallmarks in multiple types of cancer. The OXPHOS system consists of five multisubunit complexes, and mtDNA encodes 7 subunits of complex I, 1 subunit of complex III, 3 subunits of complex IV, and 2 subunits of complex V [16], [27]. Therefore, to understand the importance of mtDNA in prostate cancer progression, we determined mtDNA content in normal prostate tissue samples, benign prostate hyperplasia (BPH) tissue, prostate tumors, and white blood cells (WBCs). We observed that the median mtDNA content was reduced to ∼72% in BPH compared to normal prostate epithelial tissues in prostate cancer patients (Table 1). In addition, mtDNA content was also reduced to ∼77% in primary prostate tumors compared to normal prostate epithelial tissues. Interestingly, mtDNA content was significantly reduced in WBCs compared to normal prostate epithelial tissues or tumors (see in Table 1–7, and Figure 1 and 2). The remaining patient demographic information including age, race, staging, Gleason grading, prostate-specific antigen (PSA) status, and the number of patients involved are mentioned in Table 1.

**Table 1:** Demographic and clinical analysis of mtDNA content in prostate cancer among American men.

		Normal	BPH	Tumor	WBCs		Samples (%)	
**Samples**	N	30 (25.4%)	20 (16.9%)	38 (32.2%)	30 (25.4%)		118 (100%)	
**mtDNA**	Mean/Std/N	38.3/50.9/30	16.4/9.8/20	75.1/336/38		3.1/1.5/30		38/193/118
	Median/Min/Max	19.5/6.2/240	14/5.3/41.8	15/5.5/2091	3/1/6.2		11.7/1/2091	
**Age (yrs)**	< 60	13 (43.3%)	7 (35.0%)	17 (44.7%)	21 (70.0%)		58 (49.2%)	
	≥ 60	17 (56.7%)	13 (65.0%)	21 (55.3%)	9 (30.0%)		60 (50.8%)	
**Race**	Caucasian	14 (46.7%)	10 (50.0%)	20 (52.6%)	15 (50.0%)		59 (50.0%)	
	African American	16 (53.3%)	10 (50.0%)	18 (47.4%)	15 (50.0%)		59 (50.0%)	
**Stage**	T2	19 (63.3%)	19 (95.0%)	24 (63.2%)	25 (83.3%)		87 (73.7%)	
	T3	11 (36.7%)	1 (5.0%)	14 (36.8%)	5 (16.7%)		31 (26.3%)	
**Gleason Sum**	< 7	7 (23.3%)	8 (40.0%)	9 (23.7%)	8 (26.7%)		32 (27.1%)	
	= 7	14 (46.7%)	8 (40.0%)	16 (42.1%)	19 (63.3%)		57 (48.3%)	
	> 7	9 (30.0%)	4 (20.0%)	13 (34.2%)	3 (10.0%)		29 (24.6%)	
**Gleason Minor**	≤ 3	16 (53.3%)	9 (45.0%)	18 (47.4%)	9 (30.0%)		52 (44.1%)	
	≥ 4	14 (46.7%)	11 (55.0%)	20 (52.6%)	21 (70.0%)		66 (55.9%)	
**Gleason Major**	≤ 3	19 (63.3%)	17 (85.0%)	24 (63.2%)	27 (90.0%)		87 (73.7%)	
	≥ 4	11 (36.7%)	3 (15.0%)	14 (36.8%)	3 (10.0%)		31 (26.3%)	
**PSA**	≤ 4	7 (23.3%)	3 (15.8%)	12 (31.6%)	7 (23.3%)		29 (24.8%)	
	4–10	18 (60.0%)	15 (79%)	21 (55.3%)	19 (63.3%)		73 (62.4%)	
	> 10	5 (16.7%)	1 (5.3%)	5 (13.2%)	4 (13.3%)		15 (12.8%)	

The mtDNA content is reported as mean, median, and standard deviation. Patient demographic and clinical variables (age, race, stage, Gleason sum, Gleason minor, Gleason major, and PSA) are reported as frequencies and relative frequencies. A QQ plot (not shown) was used to assess the normality of mtDNA and these values were determined to be non-normal, with no normalizing transformation available. The percentages for age to PSA are column percentages; the percentages are derived from the total number in the first row. The percentages in the first row are the only ones based on the sample size of 118.

**Table 2:** Comparison of mtDNA content in normal, BPH, tumor, and WBCs between different stages.

	Tumor Stage	T2	T3	P-value
**Normal**	Mean/Std/N Median/Min/Max	34.6/53.6/19 19.5/8.1/240	44.6/47.8/11 28.3/6.2/148	0.641
**BPH**	Mean/Std/N Median/Min/Max	16.4/10/19 13.7/5.3/41.8	16.8/./1 16.8/16.8/16.8	0.700
**Tumor**	Mean/Std/N Median/Min/Max	22.2/19.3/24 15.2/5.5/94.8	166/554/14 14.7/6.5/2091	0.692
**WBCs**	Mean/Std/N Median/Min/Max	2.9/1.6/25 2.7/0.9/6.2	4/1.1/5 3.6/3.2/5.8	0.150

In each patient group, the mtDNA values are compared between tumor stages using Wilcoxon ranked-sum test.

**Table 3:** Comparison of mtDNA content in normal, BPH, tumor, and WBCs between different levels of PSA.

	PSA	≤ 4	4–10	>10	P-value
**Normal**	Mean/Std/N Median/Min/Max	37/42/7 10.5/7.5/120	39/59.6/18 19.5/6/240	38.3/33/5 28/9.5/93	0.689
**BPH**	Mean/Std/N Median/Min/Max	9.8/1.6/3 9.3/8.5/11.7	18/10.5/15 16.2/5.3/42	7.1/./1 7.1/7.1/7	0.103
**Tumor**	Mean/Std/N Median/Min/Max	24.1/27.5/12 14.1/5.5/95	118/452/21 16/6.5/2091	18.6/18/5 11/6.5/50	0.429
**WBCs**	Mean/Std/N Median/Min/Max	3.4/2/7 2.6/1.2/5.9	3/1.4/19 3.2/0.9/6.2	3.3/1.3/4 2.9/2.1/5	0.909

In each patient group, the mtDNA values are compared between PSA levels using Kruskal-Wallis exact test.

**Table 4:** Comparison of mtDNA content in normal, BPH, tumor, and WBCs between different Gleason grades (Gleason Sum).

Gleason Sum		< 7	= 7	> 7	P-value
**Normal**	Mean/Std/N	19/9/7	46.3/69/14	41/34.4/9	
	Median/Min/Max	19.5/7.5/30	11.3/8/240	41/6.2/120	0.466
**BPH**	Mean/Std/N	15.4/11/8	16.4/5/8	18.6/16/4	
	Median/Min/Max	12.3/5.3/39	15.4/12/26	12.6/7/42	0.570
**Tumor**	Mean/Std/N	20.6/10.5/9	152/518/16	17.8/15/13	
	Median/Min/Max	21/7.5/43.9	15/5.5/2091	13/6.5/66	0.491
**WBCs**	Mean/Std/N	3.5/1.3/8	3/1.7/19	2.8/0.6/3	
	Median/Min/Max	3.1/2.2/5.9	2.7/1/6.2	3.2/2.1/3	0.669

In each patient group, mtDNA values are compared between the Gleason grades (Gleason Sum) using Kruskal-Wallis exact test.

**Table 5:** Comparison of mtDNA content in normal, BPH, tumor, and WBCs between different Gleason grades (Gleason Minor).

Gleason Minor		≤ 3	≥ 4	P-value
**Normal**	Mean/Std/N	34.5/37.7/16	42.6/64.1/14	
	Median/Min/Max	22.7/7.5/147.5	15.3/6.2/240	0.918
**BPH**	Mean/Std/N	16.6/11/9	16.3/9.3/11	
	Median/Min/Max	13.7/5.3/39	14/7.1/41.8	0.766
**Tumor**	Mean/Std/N	135/488/18	21/22.4/20	
	Median/Min/Max	19.2/6.5/2091	13.6/5.5/94.8	0.143
**WBCs**	Mean/Std/N	3.4/1.2/9	3/1.7/21	
	Median/Min/Max	3.2/2.2/5.9	2.7/0.9/6.2	0.377

In each patient group, mtDNA values are compared between the Gleason grades (Gleason Minor) using Wilcoxon ranked-sum test.

**Table 6:** Comparison of mtDNA content in normal, BPH, tumor, and WBCs between different Gleason grades (Gleason Major).

Gleason Major		≤ 3	≥ 4	P-value
**Normal**	Mean/Std/N	32.4/51.6/19	48.5/50.5/11	
	Median/Min/Max	19.5/7.5/240	28.3/6.2/147.5	0.735
**BPH**	Mean/Std/N	16.4/10/17	16.7/9.6/3	
	Median/Min/Max	13.7/5.3/41.8	16.8/7.1/26.3	0.765
**Tumor**	Mean/Std/N	20.5/18.6/24	168.7/553.6/14	
	Median/Min/Max	15.2/5.5/94.8	15.1/6.5/2091	0.782
**WBCs**	Mean/Std/N	3.1/1.6/27	2.8/0.6/3	
	Median/Min/Max	2.8/0.9/6.2	3.2/2.1/3.2	0.800

In each patient group, mtDNA values are compared between the Gleason grades (Gleason Major) using Wilcoxon ranked-sum test.

**Table 7:** Comparison of mtDNA in normal, tumor, BPH, and WBCs between race after adjustment with age, Gleason, and PSA.

		CA	AA	Race P-value	Factor P-value	Interaction P-value
**Normal**	Age Adjusted	46.9 (14.4)	30.9 (12.9)	0.565	0.624	0.563
	Gleason Adjusted	42.8 (16.5)	28.9 (13.9)	0.555	0.432	0.294
	PSA Adjusted	47.7 (15.4)	25.6 (17.4)	0.523	0.234	0.454
**BPH**	Age Adjusted	15.4 (3.2)	19.7 (3.4)	0.565	0.519	0.588
	Gleason Adjusted	15.5 (4.6)	18.1 (3.8)	0.682	0.301	0.389
	*PSA Adjusted	. (.)	12.5 (4.3)	0.767	0.587	0.627
**Tumor**	Age Adjusted	150 (76)	20.6 (78.5)	0.498	0.504	0.634
	Gleason Adjusted	98 (86.9)	20 (82.2)	0.549	0.196	0.210
	PSA Adjusted	90.3 (88.1)	19.5 (103)	0.664	0.197	0.259
**WBCs**	Age Adjusted	3.2 (0.4)	2.7 (0.5)	0.540	0.522	0.572
	Gleason Adjusted	3.2 (0.6)	3.06 (0.53)	0.773	0.153	0.453
	PSA Adjusted	3.4 (0.5)	3.11 (0.52)	0.764	0.335	0.725

Adjusted mtDNA analyses were performed using two-way ANOVA, with the appropriate p-values obtained using standard bootstrap methodologies to account for the non-normal mtDNA values. For each patient group, the adjusted mean mtDNA values are presented by race (standard error presented in parentheses). “Race p-value” corresponds to the adjusted comparison of race; “factor p-value” corresponds to the effect of age, Gleason sum, or PSA on mtDNA; “interaction p-value” corresponds to testing for an interaction effect (i.e., whether the difference between race depend on the age, Gleason sum or PSA level). *There is no average for the CA in the PSA adjusted model because there is no CA with PSA >10 in the BPH.

### Prostate Cancer Patients Over 60 Years of Age Harbor Reduced mtDNA Content in Tumors but Increased mtDNA Content in Normal Prostate Tissues

Age comparison of mtDNA content in prostate cancer patients demonstrated that in normal prostate epithelial tissues, median mtDNA content increased ∼229% in patients ≥60 years compared to patients <60 years of age. Similarly, mean values were also increased in patients ≥60 years of age (Figure 1A and B). In contrast to normal prostate epithelial tissues, mtDNA content was reduced in both BPH and tumor tissues obtained from patients ≥60 years of age compared to patients aged <60 years. Although the mean mtDNA content in tumors was ∼7 fold lower in patients aged ≥60 versus <60 years of age, the median values showed only ∼16% reduction in older prostate cancer patients (Figure 1A and B). It is interesting to note that mtDNA content in tumor samples showed high variability, which is consistent with earlier findings that tumor cells show high levels of mtDNA variability [16]. The levels of mtDNA in WBCs were also reduced in patients ≥60 years of age (Figure 1A).

### Primary Prostate Tumors and Normal Prostate Tissues from AA Men Possess Reduced mtDNA Content Compared to CA Men

Prostate cancer is highly aggressive in AA men compared to CA men; however, the underlying mechanisms are not yet defined. Decreased mtDNA content is one of the key factors in cancer progression, including in prostate cancer [7], [25], [26]. However, comparative analyses of mtDNA between AA and CA patients have not been attempted. To understand why AA men show higher incidence and aggressiveness of prostate cancer compared to CA men, we analyzed mtDNA content in these two groups of prostate cancer patients. We observed that in normal prostate epithelial tissues, the levels of mtDNA in AA men were significantly lower (54%) compared to CA men (p = 0.047). Although the mean mtDNA content in prostate tumor tissues from AA men was 6-fold lower compared to CA men, the median mtDNA content showed only ∼16% reduction. Similarly, mtDNA content was also reduced to ∼73% in BPH from AA compared to CA patients (Figure 1C and D). WBCs from these two groups of patients did not show any significant changes in mtDNA content (Figure 1C).

### Higher mtDNA Levels in Normal Tissues Correspond to Higher Staging in Prostate Cancer

To further establish and evaluate the importance of mtDNA in cancer progression, we compared different prostate cancer stages in both groups of combined populations. Surprisingly, we observed higher mtDNA content in matched normal tissues at the T3 stage (i.e., tumor spread through prostatic capsule) compared to the T2 stage (i.e., benign stage). Because T3 stage tumors have worse prognoses compared to the T2 stage, mtDNA in normal tissues from T3 tumors needs further investigation. Although the reason for increased mtDNA in normal tissues from tumors of higher grade and stage is not known, one plausible explanation might be due to accumulation of an increased number of altered or defective mitochondria in normal cells that were not transformed, thus causing increased amount of mtDNA in normal tissues at higher grade and stage. Interestingly, mtDNA content was not affected in tumor tissues or in BPH samples. Importantly, increased mtDNA levels in WBCs at the T3 stage suggest that mtDNA content could also be a possible marker for staging and predicting aggressiveness of prostate cancer (Table 2).

Analyses of PSA levels and mtDNA content demonstrated that median mtDNA content was positively associated with PSA levels in normal tissues, whereas it was decreased in tumors from patients with PSA levels >10. No specific trend was observed in BPH samples (Table 3).

### mtDNA Content Inversely Relates with Gleason Grade in Prostate Cancer Patients

With regard to Gleason grade, mtDNA content in normal prostate epithelial tissues was nearly doubled in Gleason grade of >7 compared with Gleason grade ≤7. In contrast, prostate tumors showed reduced median values of mtDNA in samples with Gleason grade ≥7; however, no difference in mtDNA content between different Gleason grades was observed in BPH samples (Table 4).

When we compared median values of mtDNA content in tumors between Gleason grade ≤3 and ≥4, we observed ∼30% reduction in mtDNA in Gleason minor grade ≥4 compared to grade ≤3, whereas no change in median mtDNA content was observed in tumors of Gleason major. Increased mtDNA levels were observed in Gleason grade ≥4 in matched normal prostate epithelial tissues of Gleason major; however, the median mtDNA levels were decreased in matched normal tissues of ≥4 in Gleason minor (Table 5 and 6).

### Differential Trends of mtDNA Content in Prostate Tumors and Normal Prostate Tissues between AA and CA Men

In order to establish the importance of mtDNA in dissecting prostate cancer health disparities, it is essential to measure the levels of mtDNA in normal noncancerous prostate epithelial tissues as well as tumor tissues obtained from AA and CA prostate cancer patients. We observed that mtDNA content was reduced in tumor tissues compared to matched normal prostate tissues in combined data from both AA and CA patients (p = 0.053). In CA prostate cancer patients, mtDNA content was 42% lower in tumor tissues compared to matched normal prostate epithelial tissues (p = 0.068). In AA prostate cancer patients, the mtDNA levels in tumors showed limited changes compared to matched normal tissues when we compared the median values. However, the mean mtDNA content was reduced in tumor tissues compared to matched normal prostate tissues. Nonetheless, mtDNA content in tumors from AA men was lower than those from CA men (Figure 2).

### BPH and WBCs Show Reduced mtDNA Levels Compared to Normal Prostate and Tumor Tissues

Analyses of mtDNA content in combined populations of prostate cancer patients demonstrated significantly lower levels of mtDNA in BPH and WBCs compared with normal prostate tissues (p = 0.001). Similarly, mtDNA content was reduced in BPH samples and WBCs in comparison with normal tissues in AA and CA patients individually (Figure 2). Comparison of median mtDNA content between BPH/tumors and WBCs showed significant differences in the mtDNA levels. Similarly, CA and AA patients also demonstrated significant changes in the mtDNA levels in tumor/BPH and WBC samples. mtDNA levels were similar in WBCs between AA and CA patients (Figure 2).

### AA Men Possess Reduced mtDNA Content Both in Normal and Prostate Tumor Tissues Compared to CA Men after Adjusting for Age, Gleason Grade, and PSA

When we compared mtDNA content in tumors obtained from AA and CA patients after adjusting for age or Gleason grade, or PSA, although not statistically significant, we observed a ∼7–8, ∼5, or 4–5 fold decrease in mtDNA, respectively, in AA patients. Age, Gleason grade, and PSA adjustment of BPH demonstrated no changes in the mtDNA levels in BPH from AA patients compared to CA patients (Table 7). Similarly, no trends were observed in WBC samples between AA and CA men. Age, Gleason, and PSA adjustment of normal matched prostate tissues also demonstrated ∼34%, 32%, and 46% decrease, respectively, in mtDNA levels in normal prostate tissues from AA patients compared to CA patients (Table 7). These data suggest that AA patients possess lower levels of mtDNA in normal prostate tissues as well as in tumor tissues, which could be a contributing factor for the higher incidence and/or aggressiveness of prostate cancer in AA men. The significance levels of pairwise comparisons between normal prostate tissues, BPH, WBCs, and tumors are also shown in Table 8.

**Table 8:** Pairwise mtDNA comparison in normal, tumor, BPH, and WBCs.

		Normal	BPH	WBCs	Tumor
**Overall**	Normal	–	0.152	<.001	0.053
	BPH		–	<.001	0.433
	Tumor				–
	WBCs			–	<.001
**CA Only**	Normal	–	0.048	<.001	0.068
	BPH		–	<.001	0.373
	Tumor				–
	WBCs			–	<.001
**AA Only**	Normal	–	0.737	<.001	0.404
	BPH		–	<.001	0.768
	Tumor				–
	WBCs			–	<.001

The median mtDNA values are compared between each patient group in a pairwise fashion using the Wilcoxon signed-rank (for normal vs tumor) or Wilcoxon rank-sum exact tests for overall samples and within the two racial sub-samples. P-values are presented here, whereas median mtDNA values for each are presented in Figure 2A.

## Discussion

Our study provides the first evidence that prostate tumor tissues from AA men harbor reduced mtDNA levels compared to CA men. Normal prostate epithelial tissues from AA men also show decreased mtDNA content as compared to CA men. Although the exact significance of these results requires further investigation, our data suggest that reduced mtDNA levels in AA men may be associated with the higher incidence and aggressive nature of prostate cancer in AA men. Future studies with increased numbers of DNA samples from AA and CA patients may provide greater understanding regarding the impact of reduced mtDNA content on prostate cancer health disparities. It is unknown how mtDNA content decreases in prostate tumors from AA men compared to CA men, but it is possible that mtDNA polymerase γ (pol γ), the only DNA polymerase in mitochondria that plays an essential role in mtDNA replication and repair [28], may not be fully functional in normal prostate epithelial and cancer cells from AA men. It is also known that p53 regulates the integrity of mtDNA [29] through its interaction with pol γ [30]. Although p53 mutations are rare in primary prostate cancer, higher tumor stage and grade, androgen-independent, and metastatic prostate cancer are associated with high level of p53 mutations [31]–[33]. Mitochondrial dysfunction is associated with the suppression of p53 activity [34] and p53 regulates mitochondrial respiration [35], [36]. These findings suggest that reduced mtDNA levels in normal prostate epithelial and cancer cells from AA men may be associated with defects in pol γ and p53 functions.

Because the occurrence of prostate cancer in AA men is higher than in CA men, as well as prostate cancer is more aggressive in AA men [1]–[3], reduced mtDNA content in normal prostate epithelial cells and tumor cells from AA men suggests that decreased mtDNA content could be one of the contributing factors for prostate cancer disparities in American men. Human mtDNA encodes 13 protein subunits of the mitochondrial respiratory chain, 22 transfer RNAs, and 2 ribosomal RNAs [27]. Thus, reduced mtDNA content may cause the inhibition of mitochondrial respiration due to lack of functional OXPHOS [27], [37]. Lack of respiration results in reduced oxygen consumption leading to the degradation of 3-hydroxy-3-methyl-glutaryl-CoA reductase (HMGR) that could ultimately contribute to maturation of the Ras oncogene [38]. Activation of Ras results in constitutive activation of multiple survival signaling pathways such as ERK, AKT, and NF-*k*B. Therefore, reduced mtDNA in normal cells from AA men suggests that the AA population is susceptible to a higher incidence of prostate cancer. Indeed, the incidence of prostate cancer in AA men is ∼1.6 times greater than the CA population [1], [2]. Decreased mtDNA content in prostate epithelial cells from AA men will result in decreased OXPHOS capacity of transformed prostate epithelial cells, which favors faster growth and invasiveness [39]. Recent findings using animal models further support our conclusion that mtDNA mutations and reduced mtDNA content results in tumor development and shortens the life span of animals [40].

Reduction or depletion of mtDNA and abrogation of mitochondrial respiration inhibits apoptosis induced by multiple anticancer agents [18], [41]–[44]. We observed a reduction in mtDNA levels in tumors compared to normal prostate epithelial tissues, suggesting that prostate cancer in AA men is refractory to the anticancer agents. The relatively higher mtDNA content in CA prostate cancer cells is more likely to respond to therapy compared to AA prostate cancer cells. Increased mtDNA content is also known to provide resistance to anticancer agents such as docetaxel [21], which may be associated with low ROS due to increased activity of F_0_F_1_-ATP synthase. These effects may also be due to differences in cancer cell types. The relatively higher mtDNA content in CA patients may lead to enhanced mitochondrial function and respiration, thereby causing enhanced apoptotic cell death in CA patients compared to AA patients. In contrast, higher mtDNA content in tumors from CA prostate cancer patients may lead to proliferation of mitochondria due to induction of genes involved in mitochondrial biogenesis. This suggests that the relatively higher mtDNA content in CA prostate cancer patients could cause accumulation of abundant but morphologically altered mitochondria, which may induce apoptosis or alternate forms of programmed cancer cell death in prostate tumors. These findings are consistent with earlier reports that increased mtDNA mutations in thyroid tumors induce accumulation of defective and altered mitochondria [45], [46]. The exact mechanism of decreased mtDNA content in tumors from AA men as well as the relatively higher mtDNA content in CA men is unknown, but because cancer development is influenced by external and environmental factors in addition to genetic factors, both factors might be playing a role in the differential expression of mtDNA in normal prostate epithelial tissues and tumors from these two populations.

How is the reduction in mtDNA associated with aggressive nature of prostate cancer in AA men? Depletion of mtDNA induces prostate cancer progression [7], [24], [25]. Depletion of mtDNA prevents apoptosis and promotes cell motility through upregulation of phosphatidylinositol 3-kinase (PI3K)/Akt2 signaling [26]. Therefore, depletion of mtDNA likely blocks apoptosis, and promotes survival and motility of cancer cells via Akt2 activation. Our data indicate that Gleason grade and PSA adjusted AA prostate cancer patients are associated with depletion of mtDNA compared to CA patients, thus supporting our conclusion that mtDNA reduction is associated with the inhibition of apoptosis and invasiveness of prostate cancer in AA men. Depletion of mtDNA induces epithelial-mesenchymal transition (EMT) [8], which is an indicator of cancer progression. mtDNA depletion also reduces PARP-1 levels [25], which promotes progression of the neoplastic phenotype in prostate cancer. These findings support our conclusion that reduced mtDNA content in AA men may be associated with more aggressive prostate cancer.

Together, our findings provide the first evidence that mtDNA content is decreased in normal prostate epithelial and cancer tissues from AA men compared to CA men. These findings may have significance in predicting the differential outcomes of prostate cancer progression and treatment in American men. Because both normal prostate tissues and tumor tissues of CA men harbor higher mtDNA levels compared to AA men, we anticipate that CA men will be more likely to respond to anticancer therapy and be more susceptible to the cytotoxic effect of anticancer agents compared to AA men. Therefore, a clear understanding of the mechanisms of mtDNA imbalance in American men may provide new and efficient approaches for prostate cancer prevention and therapy.

## Materials and Methods

### Source of Tissue Specimens, Ethical Considerations, and Genomic DNA Extraction

Sample collection was performed after obtaining written informed consent from patients for the use of their samples for diagnostic and scientific purposes. All clinical samples were provided based on a unique and random alphanumerical coded system without referring to the patients by name or using any other personal identifier term. During data processing and analyses, samples were assigned a different random number. Ethical approval was obtained from the Institutional Review Boards (IRBs) at Louisiana State University Health Sciences Center (LSUHSC), School of Medicine, New Orleans, LA, and Roswell Park Cancer Institute, Buffalo, NY. The study conformed to the principles outlined in the Declaration of Helsinki. All tissue biospecimens were obtained from the biospecimen core facilities at the Louisiana Cancer Research Consortium (LCRC) affiliated with Tulane Medical School and School of Medicine, New Orleans and LSUHSC, New Orleans (http://www.louisianacancercenter.org/collaborative-initiatives/shared-resources/biospecimen-core).

Normal research subjects (the source of BPH samples) in this study are defined as men with no evidence of prostate cancer. White blood cells (WBCs) were obtained from patients diagnosed with primary prostate cancer. Fresh frozen matched normal and malignant prostate tissues were obtained from African American and Caucasian American patients diagnosed with primary tumors. In this study, normal prostate tissues are considered as matched normal prostate specimens taken from patients with primary prostate cancer. Only malignant tissues with 70% or higher tumor content were considered in the study. Genomic DNAs were extracted using the QIAGEN DNeasy Blood & Tissue Kit, as recommended by the manufacturer. Genomic DNA samples were quantitated using a NanoDrop 8000 Spectrophotometer (Thermo Scientific).

### Analyses of mtDNA Content Using Real-time PCR

mtDNA content was determined as described previously [47]. In brief, total genomic DNA (containing both mtDNA and nuclear DNA) was isolated from matched normal and prostate tumor tissues, BPH, and WBCs from AA and CA prostate cancer patients as described above. After quantification of DNA samples by the NanoDrop 8000 Spectrophotometer, mtDNA content was determined using the Applied Biosystems 7300 real-time PCR system. *β-actin* and *cytochrome c oxidase subunit II (COX II)* were used for amplification of nuclear and mtDNA, respectively. Primers for *β-actin* and *COX II* were used as described previously [47], [48] and the sequences are as follows: *β-actin* (forward): 5′-TCAC CCACACTGTGCCCATCTACGA-3′, *β-actin* (reverse): 5′-CAGCGGAACCGCTCATTGCCAATGG-3′. *COX II* (forward): 5′-CCCCACATTAGGCTTAAAAACAGAT-3′, *COX II* (reverse): 5′ TATACCCCCGGTCGTGTAGCG GT-3′. Real-time PCR reactions were performed in a total reaction volume of 10 µl containing 5 µl 2X iTaq SYBR Green Supermix with ROX (Bio-Rad, Cat# 172-5850), 10 ng of template DNA, 500 nM each of forward and reverse primers, and nuclease-free water. Melting curve analyses were performed at the end of the amplification to verify the absence of nonspecific amplification or primer dimer formation. The threshold cycle number (Ct) values for each reaction were calculated using the 7300 system SDS software. Standard curves were generated using 10 ng to 10 pg of DNA prepared from untreated LNCaP cells and provided PCR efficiency based on the equation E = 10^∧^(−1/slope) −1 [49]. Average Ct values were obtained by amplification of *COX II* (mtDNA-specific) and *β-actin (*nDNA-specific). mtDNA content was determined as 2^∧^ΔCt, or fold difference of mtDNA from nDNA [29], [47], [50].

### Statistical Analyses

mtDNA content was reported using the means, medians, and standard deviations. Other clinical and demographic characteristics were reported using frequencies and relative frequencies. Graphical analyses (QQ plots) found the mtDNA content to be non-normally distributed and therefore, non-parametric approaches were considered for analyses of this variable. For each group (normal, tumor, BPH, and WBCs), the mtDNA content was compared between age, race, tumor stage, prostate specific antigen (PSA), Gleason sum, Gleason minor and Gleason major using Wilcoxon rank-sum or Kruskal-Wallis exact tests, as appropriate. Additional analyses within each group included comparisons of mtDNA content between racial groups while adjusting for age, Gleason sum and PSA using two-way analysis of variance (ANOVA). Because mtDNA content is non-normally distributed, appropriate p-values were obtained using standard bootstrap methodologies. mtDNA content was compared in a pairwise fashion between groups in the overall sample and within each race using the Wilcoxon signed-rank (healthy vs. tumor) or Wilcoxon rank-sum (all other comparisons) exact tests. All data analyses were conducted in SAS v9.3 (Cary, NC) at a significance level of 0.05.
